# Synthesizing JIT Compilers for In-Kernel DSLs

**DOI:** 10.1007/978-3-030-53291-8_29

**Published:** 2020-06-16

**Authors:** Jacob Van Geffen, Luke Nelson, Isil Dillig, Xi Wang, Emina Torlak

**Affiliations:** 8 Microsoft Research Lab, Redmond, WA USA; 9 University of Southern California, Los Angeles, CA USA; 10 University of Washington, Seattle, USA; 11 University of Texas at Austin, Austin, USA

**Keywords:** Synthesis, Just-in-time compilation, Symbolic execution

## Abstract

Modern operating systems allow user-space applications to submit code for kernel execution through the use of in-kernel domain specific languages (DSLs). Applications use these DSLs to customize system policies and add new functionality. For performance, the kernel executes them via just-in-time (JIT) compilation. The correctness of these JITs is crucial for the security of the kernel: bugs in in-kernel JITs have led to numerous critical issues and patches.

This paper presents JitSynth, the first tool for synthesizing verified JITs for in-kernel DSLs. JitSynth takes as input interpreters for the source DSL and the target instruction set architecture. Given these interpreters, and a mapping from source to target states, JitSynth synthesizes a verified JIT compiler from the source to the target. Our key idea is to formulate this synthesis problem as one of synthesizing a per-instruction compiler for *abstract register machines*. Our core technical contribution is a new *compiler metasketch* that enables JitSynth to efficiently explore the resulting synthesis search space. To evaluate JitSynth, we use it to synthesize a JIT from eBPF to RISC-V and compare to a recently developed Linux JIT. The synthesized JIT avoids all known bugs in the Linux JIT, with an average slowdown of $1.82\times$ in the performance of the generated code. We also use JitSynth to synthesize JITs for two additional source-target pairs. The results show that JitSynth offers a promising new way to develop verified JITs for in-kernel DSLs.

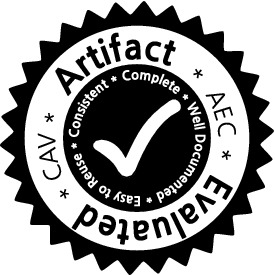



## Introduction

Modern operating systems (OSes) can be customized with user-specified programs that implement functionality like system call whitelisting, performance profiling, and power management [11, 12, 24]. For portability and safety, these programs are written in restricted domain-specific languages (DSLs), and the kernel executes them via interpretation and, for better performance, just-in-time (JIT) compilation. The correctness of in-kernel interpreters and JITs is crucial for the reliability and security of the kernel, and bugs in their implementations have led to numerous critical issues and patches [15, 30]. More broadly, embedded DSLs are also used to customize—and compromise [6, 18]—other low-level software, such as font rendering and anti-virus engines [8]. Providing formal guarantees of correctness for in-kernel DSLs is thus a pressing practical and research problem with applications to a wide range of systems software.

Prior work has tackled this problem through interactive theorem proving. For example, the Jitk framework [40] uses the Coq interactive theorem prover [38] to implement and verify the correctness of a JIT compiler for the classic Berkeley Packet Filter (BPF) language [24] in the Linux kernel. But such an approach presents two key challenges. First, Jitk imposes a significant burden on DSL developers, requiring them to implement both the interpreter and the JIT compiler in Coq, and then manually prove the correctness of the JIT compiler with respect to the interpreter. Second, the resulting JIT implementation is extracted from Coq into OCaml and cannot be run in the kernel; rather, it must be run in user space, sacrificing performance and enlarging the trusted computing base (TCB) by relying on the OCaml runtime as part of the TCB.

This paper addresses these challenges with JitSynth, the first tool for synthesizing verified JIT compilers for in-kernel DSLs. JitSynth takes as input interpreters for the source DSL and the target instruction set architecture (ISA), and it synthesizes a JIT compiler that is guaranteed to transform each source program into a semantically equivalent target program. Using JitSynth, DSL developers write no proofs or compilers. Instead, they write the semantics of the source and target languages in the form of interpreters and a mapping from source to target states, which JitSynth trusts to be correct. The synthesized JIT compiler is implemented in C; thus, it can run directly in the kernel.

At first glance, synthesizing a JIT compiler seems intractable. Even the simplest compiler contains thousands of instructions, whereas existing synthesis techniques scale to tens of instructions. To tackle this problem in our setting, we observe that in-kernel DSLs are similar to ISAs: both take the form of bytecode instructions for an *abstract register machine*, a simple virtual machine with a program counter, a few registers, and limited memory store [40]. We also observe that in practice, the target machine has at least as many resources (registers and memory) as the source machine; and that JIT compilers for such abstract register machines perform register allocation statically at compile time. Our main insight is that we can exploit these properties to make synthesis tractable through *decomposition* and *prioritization*, while preserving soundness and completeness.

JitSynth works by decomposing the JIT synthesis problem into the problem of synthesizing individual *mini compilers* for every instruction in the source language. Each mini compiler is synthesized by generating a *compiler metasketch* [7], a set of ordered sketches that collectively represent *all* instruction sequences in the target ISA. These sketches are then solved by an off-the-shelf synthesis tool based on reduction to SMT [39]. The synthesis tool ensures that the target instruction sequence is semantically equivalent to the source instruction, according to the input interpreters. The order in which the sketches are explored is key to making this search practical, and JitSynth contributes two techniques for biasing the search towards tightly constrained, and therefore tractable, sketches that are likely to contain a correct program.

First, we observe that source instructions can often be implemented with target instructions that access the same parts of the state (e.g., only registers). Based on this observation, we develop *read-write sketches*, which restrict the synthesis search space to a subset of the target instructions, based on a sound and precise summary of their semantics. Second, we observe that hand-written JITs rely on pseudoinstructions to generate common target sequences, such as loading immediate (constant) values into registers. We use this observation to develop *pre-load sketches*, which employ synthesized pseudoinstructions to eliminate the need to repeatedly search for common target instruction subsequences.

We have implemented JitSynth in Rosette [39] and used it to synthesize JIT compilers for three widely used in-kernel DSLs. As our main case study, we used JitSynth to synthesize a RISC-V [32] compiler for extended BPF (eBPF) [12], an extension of classic BPF [24], used by the Linux kernel. Concurrently with our work, Linux developers manually built a JIT compiler for the same source and target pair, and a team of researchers found nine correctness bugs in that compiler shortly after its release [28]. In contrast, our JIT compiler is verified by construction; it supports 87 out of 102 eBPF instructions and passes all the Linux kernel tests within this subset, including the regression tests for these nine bugs. Our synthesized compiler generates code that is $5.24\times$ faster than interpreted code and $1.82\times$ times slower than the code generated by the Linux JIT. We also used JitSynth to synthesize a JIT from libseccomp [10], a policy language for system call whitelisting, to eBPF, and a JIT from classic BPF to eBPF. The synthesized JITs avoid previously found bugs in the existing generators for these source target pairs, while incurring, on average, a $2.28$–$2.61\times$ slowdown in the performance of the generated code.

To summarize, this paper makes the following contributions:

1. JitSynth, the first tool for synthesizing verified JIT compilers for in-kernel DSLs, given the semantics of the source and target languages as interpreters.
2. A novel formulation of the JIT synthesis problem as one of synthesizing a per-instruction compiler for *abstract register machines*.
3. A novel *compiler metasketch* that enables JitSynth to solve the JIT synthesis problem with an off-the-shelf synthesis engine.
4. An evaluation of JitSynth ’s effectiveness, showing that it can synthesize verified JIT compilers for three widely used in-kernel DSLs.

The rest of this paper is organized as follows. Section 2 illustrates JitSynth on a small example. Section 3 formalizes the JIT synthesis problem for in-kernel DSLs. Section 4 presents the JitSynth algorithm for generating and solving compiler metasketches. Section 5 provides implementation details. Section 6 evaluates JitSynth. Section 7 discusses related work. Section 8 concludes.

**Fig. 1.**
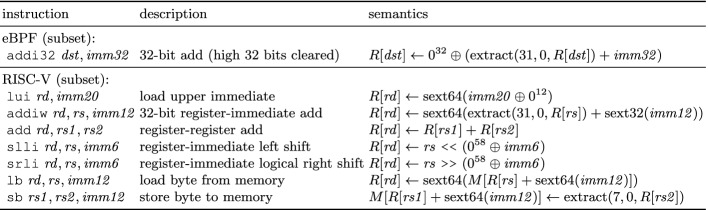
Subsets of eBPF and RISC-V used as source and target languages, respectively, in our running example: *R*[*r*] denotes the value of register *r*; *M*[*a*] denotes the value at memory address *a*; $\oplus$ denotes concatenation of bitvectors; superscripts (e.g., $0^{32}$) denote repetition of bits; $sext32(x)$ and $sext64(x)$ sign-extend *x* to 32 and 64 bits, respectively; and ${{\,\mathrm{extract}\,}}(i, j, x)$ produces a subrange of bits of *x* from index *i* down to *j*.

## Overview

This section provides an overview of JitSynth by illustrating how it synthesizes a toy JIT compiler (Fig. 1). The source language of the JIT is a tiny subset of eBPF [12] consisting of one instruction, and the target language is a subset of 64-bit RISC-V [32] consisting of seven instructions. Despite the simplicity of our languages, the Linux kernel JIT used to produce incorrect code for this eBPF instruction [27]; such miscompilation bugs not only lead to correctness issues, but also enable adversaries to compromise the OS kernel by crafting malicious eBPF programs [40]. This section shows how JitSynth can be used to synthesize a JIT that is verified with respect to the semantics of the source and target languages.

*In-Kernel Languages.* JitSynth expects the source and target languages to be a set of instructions for manipulating the state of an *abstract register machine* (Sect. 3). This state consists of a program counter ($pc$), a finite sequence of general-purpose registers ($reg$), and a finite sequence of memory locations ($mem$), all of which store bitvectors (i.e., finite precision integers). The length of these bitvectors is defined by the language; for example, both eBPF and RISC-V store 64-bit values in their registers. An instruction consists of an *opcode* and a finite set of *fields*, which are bitvectors representing either register identifiers or immediate (constant) values. For instance, the addi32 instruction in eBPF has two fields: $dst$ is a 4-bit value representing the index of the output register, and $imm32$ is a 32-bit immediate. (eBPF instructions may have two additional fields $src$ and $off$, which are not shown here as they are not used by addi32). An abstract register machine for a language gives meaning to its instructions: the machine consumes an instruction and a state, and produces a state that is the result of executing that instruction. Figure 1 shows a high-level description of the abstract register machines for our languages.

**Fig. 2.**
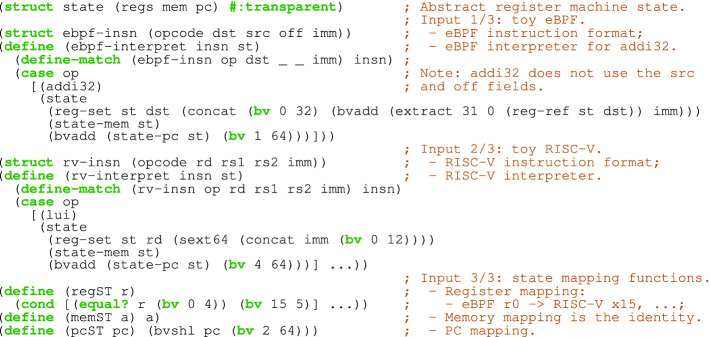
Snippets of inputs to JitSynth: the interpreters for the source (eBPF) and and target (RISC-V) languages and state-mapping functions.

JitSynth *Interface.* To synthesize a compiler from one language to another, JitSynth takes as input their syntax, semantics, and a mapping from source to target states. All three inputs are given as a program in a *solver-aided host language* [39]. JitSynth uses Rosette as its host, but the host can be any language with a symbolic evaluation engine that can reduce the semantics of host programs to SMT constraints (e.g., [37]). Figure 2 shows the interpreters for the source and target languages (i.e., emulators for their abstract register machines), as well as the state-mapping functions regST, pcST, and memST that JitSynth uses to determine whether a source state $\sigma _S$ is equivalent to a target state $\sigma _T$. In particular, JitSynth deems these states equivalent, denoted by $\sigma _S \cong \sigma _T$, whenever $reg (\sigma _T)[\texttt {regST}(r)] = reg (\sigma _S)[r]$, $pc (\sigma _T) = \texttt {pcST}( pc (\sigma _S))$, and $mem (\sigma _T)[\texttt {memST}(a)] = mem (\sigma _S)[a]$ for all registers *r* and memory addresses *a*.

*Decomposition into Per-instruction Compilers.* Given these inputs, JitSynth generates a *per-instruction compiler* from the source to the target language. To ensure that the resulting compiler is correct (Theorem 1), and that one will be found if it exists (Theorem 2), JitSynth puts two restrictions on its inputs. First, the inputs must be self-finitizing [39], meaning that both the interpreters and the mapping functions must have a finite symbolic execution tree when applied to symbolic inputs. Second, the target machine must have at least as many registers and memory locations as the source machine; these storage cells must be as wide as those of the source machine; and the state-mapping functions (pcST, regST, and memST) must be injective. Our toy inputs satisfy these restrictions, as do the real in-kernel languages evaluated in Sect. 6.

*Synthesis Workflow.* JitSynth generates a per-instruction compiler for a given source and target pair in two stages. The first stage uses an optimized *compiler metasketch* to synthesize a mini compiler from every instruction in the source language to a sequence of instructions in the target language (Sect. 4). The second stage then simply stitches these mini compilers into a full C compiler using a trusted outer loop and a switch statement. The first stage is a core technical contribution of this paper, and we illustrate it next on our toy example.

*Metasketches.* To understand how JitSynth works, consider the basic problem of determining if every addi32 instruction can be emulated by a sequence of *k* instructions in toy RISC-V. In particular, we are interested in finding a program $C_\texttt {addi32}$ in our host language (which JitSynth translates to C) that takes as input a source instruction $s = \texttt {addi32}\ dst , imm32$ and outputs a semantically equivalent RISC-V program $t = [t_1,\ldots ,t_k]$. That is, for all $dst , imm32$, and for all equivalent states $\sigma _S \cong \sigma _T$, we have $run (s, \sigma _S, \texttt {ebpf-interpret})\cong run (t, \sigma _T, \texttt {rv-interpret})$, where $run (e, \sigma , f)$ executes the instruction interpreter *f* on the sequence of instructions *e*, starting from the state $\sigma$ (Definition 3).

We can solve this problem by asking the host synthesizer to search for $C_\texttt {addi32}$ in a space of candidate mini compilers of length *k*. We describe this space with a syntactic template, or a *sketch*, as shown below:
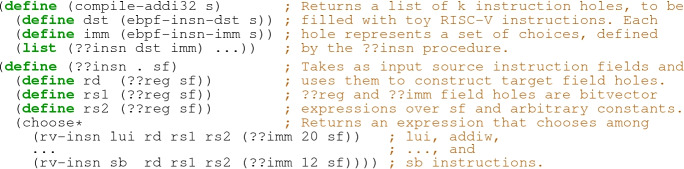


Here, (??insn dst imm) stands for a missing expression—a hole—that the synthesizer needs to fill with an instruction from the toy RISC-V language. To fill an instruction hole, the synthesizer must find an expression that computes the value of the target instruction’s fields. JitSynth limits this expression language to bitvector expressions (of any depth) over the fields of the source instruction and arbitrary bitvector constants.

Given this sketch, and our correctness specification for $C_\texttt {addi32}$, the synthesizer will search the space defined by the sketch for a program that satisfies the specification. Below is an example of the resulting toy compiler from eBPF to RISC-V, synthesized and translated to C by JitSynth (without the outer loop):
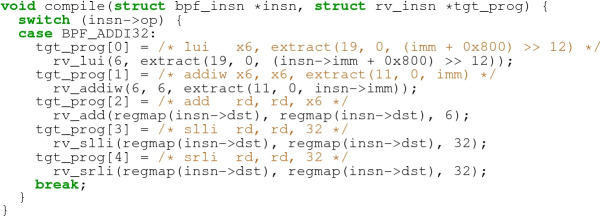


Once we know how to synthesize a compiler of length *k*, we can easily extend this solution into a naive method for synthesizing a compiler of any length. We simply enumerate sketches of increasing lengths, $k = 1, 2, 3, \ldots$, invoke the synthesizer on each generated sketch, and stop as soon as a solution is found (if ever). The resulting ordered set of sketches forms a metasketch [7]—i.e., a search space and a strategy for exploring it—that contains all candidate mini compilers (in a subset of the host language) from the source to the target language. This naive metasketch can be used to find a mini compiler for our toy example in 493 min. However, it fails to scale to real in-kernel DSLs (Sect. 6), motivating the need for JitSynth ’s optimized compiler metasketches.

*Compiler Metasketches.* JitSynth optimizes the naive metasketch by extending it with two kinds of more tightly constrained sketches, which are explored first. A constrained sketch of size *k* usually contains a correct solution of a given size if one exists, but if not, JitSynth will eventually explore the naive sketch of the same length, to maintain completeness. We give the intuition behind the two optimizations here, and present them in detail in Sect. 4.

First, we observe that practical source and target languages include similar kinds of instructions. For example, both eBPF and RISC-V include instructions for adding immediate values to registers. This similarity often makes it possible to emulate a source instruction with a sequence of target instructions that access the same part of the state (the program counter, registers, or memory) as the source instruction. For example, addi32 reads and writes only registers, not memory, and it can be emulated with RISC-V instructions that also access only registers. To exploit this observation, we introduce *read-write sets*, which summarize, soundly and precisely, how an instruction accesses state. JitSynth uses these sets to define *read-write sketches* for a given source instruction, including only target instructions that access the state in the same way as the source instruction. For instance, a read-write sketch for addi32 excludes both lb and sb instructions because they read and write memory as well as registers.

Second, we observe that hand-written JITs use pseudoinstructions to simplify their implementation of mini compilers. These are simply subroutines or macros for generating target sequences that implement common functionality. For example, the Linux JIT from eBPF to RISC-V includes a pseudoinstruction for loading 32-bit immediates into registers. JitSynth mimics the way hand-written JITs use pseudoinstructions with the help of *pre-load sketches*. These sketches first use a synthesized pseudoinstruction to create a sequence of concrete target instructions that load source immediates into scratch registers; then, they include a compute sequence comprised of read-write instruction holes. Applying these optimizations to our toy example, JitSynth finds a mini compiler for addi32 in 5 s—a roughly $6000\times$ speedup over the naive metasketch.

## Problem Statement

This section formalizes the compiler synthesis problem for in-kernel DSLs. We focus on JIT compilers, which, for our purposes, means one-pass compilers [11]. To start, we define *abstract register machines* as a way to specify the syntax and semantics of in-kernel languages. Next, we formulate our compiler synthesis problem as one of synthesizing a set of sound *mini compilers* from a single source instruction to a sequence of target instructions. Finally, we show that these mini compilers compose into a sound JIT compiler, which translates every source program into a semantically equivalent target program.

*Abstract Register Machines.* An abstract register machine (ARM) provides a simple interface for specifying the syntax and semantics of an in-kernel language. The syntax is given as a set of abstract instructions, and the semantics is given as a transition function over instructions and machine states.

An *abstract instruction* (Definition 1) defines the name ($op$) and type signature ($\mathcal {F}$) of an operation in the underlying language. For example, the abstract instruction $( addi32 , r \mapsto Reg , imm32 \mapsto BV (32))$ specifies the name and signature of the addi32 operation from the eBPF language (Fig. 1). Each abstract instruction represents the (finite) set of all *concrete instructions* that instantiate the abstract instruction’s parameters with values of the right type. For example, $\texttt {addi32}\, 0, 5$ is a concrete instantiation of the abstract instruction for addi32. In the rest of this paper, we will write “instruction” to mean a concrete instruction.

### Definition 1 (Abstract and Concrete Instructions)

An abstract instruction $$\iota $$ is a pair $$( op , \mathcal {F})$$ where $$ op $$ is an opcode and $$\mathcal {F}$$ is a mapping from *fields* to their *types*. Field types include $$ Reg $$, denoting register names, and $$ BV (k)$$, denoting *k*-bit bitvector values. The abstract instruction $$\iota $$ represents all concrete instructions $$p = ( op , F)$$ with the opcode $$ op $$ that bind each field $$f\in dom (\mathcal {F})$$ to a value *F*(*f*) of type $$\mathcal {F}(f)$$. We write $$P(\iota )$$ to denote the set of all concrete instructions for $$\iota $$, and we extend this notation to sets of abstract instructions in the usual way, i.e., $$P(\mathcal {I})=\bigcup _{\iota \in \mathcal {I}}P(\iota )$$ for the set $$\mathcal {I}$$.

Instructions operate on machine *states* (Definition 2), and their semantics are given by the machine’s *transition function* (Definition 3). A machine state consists of a program counter, a map from register names to register values, and a map from memory addresses to memory values. Each state component is either a bitvector or a map over bitvectors, making the set of all states of an ARM finite. The transition function of an ARM defines an interpreter for the ARM’s language by specifying how to compute the output state for a given instruction and input state. We can apply this interpreter, together with the ARM’s *fuel function*, to define an *execution* of the machine on a program and an initial state. The fuel function takes as input a sequence of instructions and returns a natural number that bounds the number of steps (i.e., state transitions) the machine can make to execute the given sequence. The inclusion of fuel models the requirement of in-kernel languages for all program executions to terminate [40]. It also enables us to use symbolic execution to soundly reduce the semantics of these languages to SMT constraints, in order to formulate the synthesis queries in Sect. 4.5.

### Definition 2 (State)

A state $$\sigma $$ is a tuple $$( pc , reg , mem )$$ where $$ pc $$ is a value, $$ reg $$ is a function from register names to values, and $$ mem $$ is a function from memory addresses to values. Register names, memory addresses, and all values are finite-precision integers, or bitvectors. We write $$|\sigma |$$ to denote the *size* of the state $$\sigma $$. The size $$|\sigma |$$ is defined to be the tuple $$(r, m, k_ pc , k_ reg , k_ mem )$$, where *r* is the number of registers in $$\sigma $$, *m* is the number of memory addresses, and $$k_ pc $$, $$k_ reg $$, and $$k_ mem $$ are the width of the bitvector values stored in the $$ pc $$, $$ reg $$, and $$ mem $$, respectively. Two states have the same size if $$|\sigma _i| = |\sigma _j|$$; one state is smaller than another, $$|\sigma _i| \le |\sigma _j|$$, if each element of $$|\sigma _i|$$ is less than or equal to the corresponding element of $$|\sigma _j|$$.

### Definition 3 (Abstract Register Machines and Executions)

An abstract register machine $\mathcal {A}$ is a tuple $(\mathcal {I}, \varSigma , \mathcal {T}, \varPhi )$ where $\mathcal {I}$ is a set of abstract instructions, $\varSigma$ is a set of states of the same size, $\mathcal {T} : P(\mathcal {I}) \rightarrow \varSigma \rightarrow \varSigma$ is a transition function from instructions and states to states, and $\varPhi : List (P(\mathcal {I})) \rightarrow \mathbb {N}$ is a *fuel function* from sequences of instructions to natural numbers. Given a state $\sigma _0\in \varSigma$ and a sequence of instructions $\mathbf {p}$ drawn from $P(\mathcal {I})$, we define the execution of $\mathcal {A}$ on $\mathbf {p}$ and $\sigma _0$ to be the result of applying $\mathcal {T}$ to $\mathbf {p}$ at most $\varPhi (\mathbf {p})$ times. That is, $\mathcal {A}(\mathbf {p}, \sigma _0) = run (\mathbf {p}, \sigma _0, \mathcal {T}, \varPhi (\mathbf {p}))$, where

$$ run (\mathbf {p}, \sigma , \mathcal {T}, k) = {\left\{ \begin{array}{ll} \sigma ,&{} \text {if } k = 0 \text { or } pc (\sigma ) \not \in [0, |\mathbf {p}|)\\ run (\mathbf {p}, \mathcal {T}(\mathbf {p}[ pc (\sigma )], \sigma ), \mathcal {T}, k-1), &{} \text {otherwise.} \end{array}\right. } $$

*Synthesizing JIT Compilers for ARMs.* Given a source and target ARM, our goal is to synthesize a one-pass JIT compiler that translates source programs to semantically equivalent target programs. To make synthesis tractable, we fix the structure of the JIT to consist of an outer loop and a switch statement that dispatches compilation tasks to a set of *mini compilers* (Definition 4). Our synthesis problem is therefore to find a sound mini compiler for each abstract instruction in the source machine (Definition 5).

### Definition 4 (Mini Compiler)

Let $\mathcal {A}_S = (\mathcal {I}_S, \varSigma _S, \mathcal {T}_S, \varPhi _S)$ and $\mathcal {A}_T = (\mathcal {I}_T, \varSigma _T, \mathcal {T}_T, \varPhi _T)$ be two abstract register machines, $\cong$ an equivalence relation on their states $\varSigma _S$ and $\varSigma _T$, and $C: P(\iota ) \rightarrow List (P(\mathcal {I}_T))$ a function for some $\iota \in \mathcal {I}_S$. We say that *C* is a sound mini compiler for $\iota$ with respect to $\cong$ iff


### Definition 5 (Mini Compiler Synthesis)

Given two abstract register machines $\mathcal {A}_S = (\mathcal {I}_S, \varSigma _S, \mathcal {T}_S, \varPhi _S)$ and $\mathcal {A}_T=(\mathcal {I}_T, \varSigma _T, \mathcal {T}_T, \varPhi _T)$, as well as an equivalence relation $\cong$ on their states, the mini compiler synthesis problem is to generate a sound mini compiler $C_\iota$ for each $\iota \in \mathcal {I}_S$ with respect to $\cong$.

The general version of our synthesis problem, defined above, uses an arbitrary equivalence relation $\cong$ between the states of the source and target machines to determine if a source and target program are semantically equivalent. JitSynth can, in principle, solve this problem with the naive metasketch described in Sect. 2. In practice, however, the naive metasketch scales poorly, even on small languages such as toy eBPF and RISC-V. So, in this paper, we focus on source and target ARMs that satisfy an additional assumption on their state equivalence relation: it can be expressed in terms of injective mappings from source to target states (Definition 6). This restriction enables JitSynth to employ optimizations (such as pre-load sketches described in Sect. 4.4) that are crucial to scaling synthesis to real in-kernel languages.

### Definition 6 (Injective State Equivalence Relation)

Let $\mathcal {A}_S$ and $\mathcal {A}_T$ be abstract register machines with states $\varSigma _S$ and $\varSigma _T$ such that $|\sigma _S|\le |\sigma _T|$ for all $\sigma _S\in \varSigma _S$ and $\sigma _T\in \varSigma _T$. Let $\mathcal {M}$ be a *state mapping* $(\mathcal {M}_ pc , \mathcal {M}_ reg , \mathcal {M}_ mem )$ from $\varSigma _S$ and $\varSigma _T$, where $\mathcal {M}_ pc$ multiplies the program counter of the states in $\varSigma _S$ by a constant factor, $\mathcal {M}_ reg$ is an injective map from register names in $\varSigma _S$ to those in $\varSigma _T$, and $\mathcal {M}_ mem$ is an injective map from memory addresses in $\varSigma _S$ to those in $\varSigma _T$. We say that two states $\sigma _S\in \varSigma _S$ and $\sigma _T\in \varSigma _T$ are equivalent according to $\mathcal {M}$, written $\sigma _S \cong _\mathcal {M} \sigma _T$, iff $\mathcal {M}_ pc ( pc (\sigma _S)) = pc (\sigma _T)$, $reg (\sigma _S)[r] = reg (\sigma _T)[\mathcal {M_ reg }(r)]$ for all register names $r\in dom ( reg (\sigma _S))$, and $mem (\sigma _S)[a] = mem (\sigma _T)[\mathcal {M_ mem }(a)]$ for all memory addresses $a\in dom ( mem (\sigma _S))$. The binary relation $\cong _\mathcal {M}$ is called an injective state equivalence relation on $\mathcal {A}_S$ and $\mathcal {A}_T$.

*Soundness of JIT Compilers for ARMs.* Finally, we note that a JIT compiler composed from the synthesized mini compilers correctly translates every source program to an equivalent target program. We formulate and prove this theorem using the Lean theorem prover [25].

### Theorem 1

**(Soundness of JIT compilers).** Let $\mathcal {A}_S = (\mathcal {I}_S, \varSigma _S, \mathcal {T}_S, \varPhi _S)$ and $\mathcal {A}_T=(\mathcal {I}_T, \varSigma _T, \mathcal {T}_T, \varPhi _T)$ be abstract register machines, $\cong _\mathcal {M}$ an injective state equivalence relation on their states such that $M_ pc ( pc (\sigma _S)) = N_ pc pc (\sigma _S)$, and $\{C_1,\ldots ,C_{|\mathcal {I}_S|}\}$ a solution to the mini compiler synthesis problem for $\mathcal {A}_S$, $\mathcal {A}_T$, and $\cong _\mathcal {M}$ where $\forall s\in P(\iota ).\ |C_i(s)| = N_ pc$. Let $\mathcal {C} : P(\mathcal {I}_S) \rightarrow List (P(\mathcal {I}_T))$ be a function that maps concrete instructions $s\in P(\iota )$ to the compiler output $C_\iota (s)$ for $\iota \in \mathcal {I}_S$. If $\mathbf {s} = s_1, \ldots , s_n$ is a sequence of concrete instructions drawn from $\mathcal {I}_S$, and $\mathbf {t} = \mathcal {C}(s_1)\cdot \ldots \cdot \mathcal {C}(s_n)$ where $\cdot$ stands for sequence concatenation, then $\forall \sigma _S\in \varSigma _S, \sigma _T\in \varSigma _T.\ \sigma _S \cong _\mathcal {M} \sigma _T \Rightarrow \mathcal {A}_S(\mathbf {s}, \sigma _S) \cong _\mathcal {M} \mathcal {A}_T(\mathbf {t}, \sigma _T)$.

## Solving the Mini Compiler Synthesis Problem

This section presents our approach to solving the mini compiler synthesis problem defined in Sect. 3. We employ syntax-guided synthesis [37] to search for an implementation of a mini compiler in a space of candidate programs. Our core contribution is an effective way to structure this space using a *compiler metasketch*. This section presents our algorithm for generating compiler metasketches, describes its key subroutines and optimizations, and shows how to solve the resulting sketches with an off-the-shelf synthesis engine.

### Generating Compiler Metasketches

JitSynth synthesizes mini compilers by generating and solving *metasketches* [7]. A metasketch describes a space of candidate programs using an ordered set of syntactic templates or *sketches* [37]. These sketches take the form of programs with missing expressions or *holes*, where each hole describes a finite set of candidate completions. JitSynth sketches are expressed in a *host language* $\mathcal {H}$ that serves both as the implementation language for mini compilers and the specification language for ARMs. JitSynth expects the host to provide a synthesizer for completing sketches and a symbolic evaluator for reducing ARM semantics to SMT constraints. JitSynth uses these tools to generate optimized metasketches for mini compilers, which we call *compiler metasketches*.

**Fig. 3.**
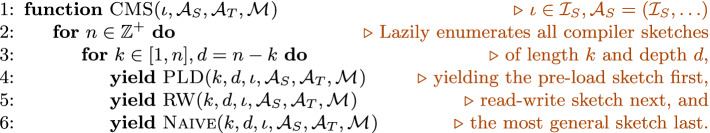
Compiler metasketch for the abstract source instruction $\iota$, source machine $\mathcal {A}_S$, target machine $\mathcal {A}_T$, and state mapping $\mathcal {M}$ from $\mathcal {A}_S$ to $\mathcal {A}_T$.

Figure 3 shows our algorithm for generating compiler metasketches. The algorithm, CMS, takes as input an abstract source instruction $\iota$ for a source machine $\mathcal {A}_S$, a target machine $\mathcal {A}_T$, and a state mapping $\mathcal {M}_{}$ from $\mathcal {A}_S$ to $\mathcal {A}_T$. Given these inputs, it lazily enumerates an infinite set of *compiler sketches* that collectively represent the space of all straight-line bitvector programs from $P(\iota )$ to $List (P(\mathcal {I}_T))$. In particular, each compiler sketch consists of *k* target *instruction holes*, constructed from field holes that denote bitvector expressions (over the fields of $\iota$) of depth *d* or less. For each length *k* and depth *d*, the CMS loop generates three kinds of compiler sketches: the *pre-load*, the *read-write*, and the *naive* sketch. The naive sketch (Sect. 4.2) is the most general, consisting of all candidate mini compilers of length *k* and depth *d*. But it also scales poorly, so CMS first yields the pre-load (Sect. 4.4) and read-write (Sect. 4.3) sketches. As we will see later, these sketches describe a subset of the programs in the naive sketch, and they are designed to prioritize exploring small parts of the search space that are likely to contain a correct mini compiler for $\iota$, if one exists.

### Generating Naive Sketches

**Fig. 4.**
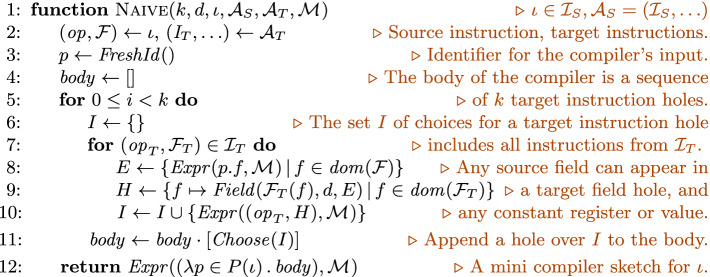
Naive sketch of length *k* and maximum depth *d* for $$\iota $$, $$\mathcal {A}_S$$, $$\mathcal {A}_T$$, and $$\mathcal {M}_{}$$. Here, $$ Expr $$ creates an expression in the host language, using $$\mathcal {M}_{}$$ to map from source to target register names and memory addresses; $$ Choose (E)$$ is a hole that chooses an expression from the set *E*; and $$ Field (\tau ,d,E)$$ is a hole for a bitvector expression of type $$\tau $$ and maximum depth *d*, constructed from arbitrary bitvector constants and expressions *E*.

The most general sketch we consider, $$\textsc {Naive} (k,d,\iota ,\mathcal {A}_S,\mathcal {A}_T,\mathcal {M}_{})$$, is shown in Fig. 4. This sketch consists of *k* instruction holes that can be filled with any instruction from $$\mathcal {I}_T$$. An instruction hole chooses between expressions of the form $$( op _T, H)$$, where $$ op _T$$ is a target opcode, and *H* specifies the field holes for that opcode. Each field hole is a bitvector expression (of depth *d*) over the fields of the input source instruction and arbitrary bitvector constants. This lets target instructions use the immediates and registers (modulo $$\mathcal {M}_{}$$) of the source instruction, as well as arbitrary constant values and register names. Letting field holes include constant register names allows the synthesized mini compilers to use target registers unmapped by $$\mathcal {M}_{}$$ as temporary, or scratch, storage. In essence, the naive sketch describes all straight-line compiler programs that can make free use of standard C arithmetic and bitwise operators, as well as scratch registers.

The space of such programs is intractably large, however, even for small inputs. For instance, it includes at least $2^{350}$ programs of length $k=5$ and depth $d\le 3$ for the toy example from Sect. 2. JitSynth therefore employs two effective heuristics to direct the exploration of this space toward the most promising candidates first, as defined by the read-write and pre-load sketches.

### Generating Read-Write Sketches

The read-write sketch, $\textsc {RW} (k, d, \iota , \mathcal {A}_S, \mathcal {A}_T, \mathcal {M}_{})$, is based on the observation that many practical source and target languages provide similar functionality, so a source instruction $\iota$ can often be emulated with target instructions that access the same parts of the state as $\iota$. For example, the addi32 instruction from eBPF reads and writes only registers (not, e.g., memory), and it can be emulated with RISC-V instructions that also touch only registers (Sect. 2). Moreover, note that the semantics of addi32 ignores the values of its $src$ and $off$ fields, and that the target RISC-V instructions do the same. Based on these observations, our optimized sketch for addi32 would therefore consists of instruction holes that allow only register-register instructions, with field holes that exclude $src$ and $off$. We first formalize this intuition with the notion of *read and write sets*, and then describe how JitSynth applies such sets to create RW sketches.

**Fig. 5.**
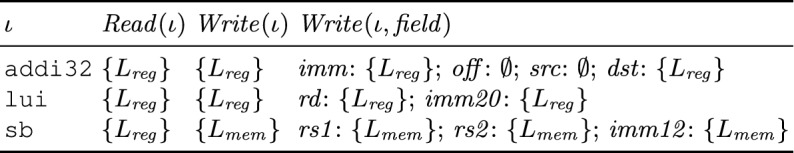
Read and write sets for the addi32, lui, and sb instructions from Fig. 1.

*Read and Write Sets.* Read and write sets provide a compact way to summarize the semantics of an abstract instruction $$\iota $$. This summary consists of a set of *state labels*, where a state label is one of $$L_ reg $$, $$L_ mem $$, and $$L_ pc $$ (Definition 7). Each label in a summary set represents a state component (registers, memory, or the program counter) that a concrete instance of $$\iota $$ may read or write during some execution. We compute three such sets of labels for every $$\iota $$: the read set $$ Read (\iota )$$, the write set $$ Write (\iota )$$, and the write set $$ Write (\iota , f)$$ for each field *f* of $$\iota $$. Figure 5 shows these sets for the toy eBPF and RISC-V instructions.

The read set $$ Read (\iota )$$ specifies which components of the input state may affect the execution of $$\iota $$ (Definition 8). For example, if $$ Read (\iota )$$ includes $$L_ reg $$, then some concrete instance of $$\iota $$ produces different output states when executed on two input states that differ only in register values. The write set $$ Write (\iota )$$ specifies which components of the output state may be affected by executing $$\iota $$ (Definition 9). In particular, if $$ Write (\iota )$$ includes $$L_ reg $$ (or $$L_ mem $$), then executing some concrete instance of $$\iota $$ on an input state produces an output state with different register (or memory) values. The inclusion of $$L_ pc $$ is based on a separate condition, designed to distinguish jump instructions from fall-through instructions. Both kinds of instructions change the program counter, but fall-through instructions always change it in the same way. So, $$L_ pc \in Write (\iota )$$ if two instances of $$\iota $$ can write different values to the program counter. Finally, the field write set, $$ Write (\iota , f)$$, specifies the parts of the output state are affected by the value of the field *f*; $$L_n \in Write (\iota , f)$$ means that two instances of $$\iota $$ that differ only in *f* can produce different outputs when applied to the same input state.

JitSynth computes all read and write sets from their definitions, by using the host symbolic evaluator to reduce the reasoning about instruction semantics to SMT queries. This reduction is possible because we assume that all ARM interpreters are self-finitizing, as discussed in Sect. 2.

#### Definition 7 (State Labels)

A *state label* is an identifier $L_n$ where *n* is a state component, i.e., $n\in \{ reg , mem , pc \}$. We write *N* for the set of all state components, and $\mathcal {L}$ for the set of all state labels. We also use state labels to access the corresponding state components: $L_n(\sigma ) = n(\sigma )$ for all $n\in N$.

#### Definition 8 (Read Set)

Let $\iota \in \mathcal {I}$ be an abstract instruction in $(\mathcal {I}, \varSigma , \mathcal {T}, \varPhi )$. The read set of $\iota$, $Read (\iota )$, is the set of all state labels $L_n\in \mathcal {L}$ such that $\exists p\in P(\iota ).\, \exists L_w \in Write (\iota ).\, \exists \sigma _a, \sigma _b \in \varSigma .\, (L_n(\sigma _a) \ne L_n(\sigma _b) \wedge (\bigwedge _{m\in N \setminus \{ n \}} L_m(\sigma _a) = L_m(\sigma _b)) \wedge L_w(\mathcal {T}(p,\sigma _a)) \ne L_w(\mathcal {T}(p,\sigma _b)).$

#### Definition 9 (Write Set)

Let $$\iota \in \mathcal {I}$$ be an abstract instruction in $$(\mathcal {I}, \varSigma , \mathcal {T}, \varPhi )$$. The write set of $$\iota $$, $$ Write (\iota )$$, includes the state label $$L_n \in \{ L_ reg , L_ mem \}$$ iff $$ \exists p\in P(\iota ).\, \exists \sigma \in \varSigma .\, L_n(\sigma ) \ne L_n(\mathcal {T}(p,\sigma )) $$, and it includes the state label $$L_ pc $$ iff $$ \exists p_a, p_b\in P(\iota ).\, \exists \sigma \in \varSigma .\, L_ pc (\mathcal {T}(p_a,\sigma )) \ne L_ pc (\mathcal {T}(p_b,\sigma )). $$

#### Definition 10 (Field Write Set)

Let *f* be a field of an abstract instruction $\iota = ( op ,\mathcal {F})$ in $(\mathcal {I}, \varSigma , \mathcal {T}, \varPhi )$. The write set of $\iota$ and *f*, $Write (\iota , f)$, includes the state label $L_n \in \mathcal {L}$ iff $\exists p_a, p_b\in P(\iota ).\, \exists \sigma \in \varSigma .\ (p_a.f \ne p_b.f) \wedge (\bigwedge _{g\in dom (\mathcal {F})\setminus \{f\}} p_a.g = p_b.g) \wedge L_n(\mathcal {T}(p_a,\sigma )) \ne L_n(\mathcal {T}(p_b,\sigma ))$, where *p*.*f* denotes *F*(*f*) for $p = (op, F)$.

*Using Read and Write Sets.* Given the read and write sets for a source instruction $\iota$ and target instructions $\mathcal {I}_T$, JitSynth generates the RW sketch of length *k* and depth *d* by modifying the Naive algorithm (Fig. 4) as follows. First, it restricts each target instruction hole (line 7) to choose an instruction $\iota _T\in \mathcal {I}_T$ with the same read and write sets as $\iota$, i.e., $Read (\iota ) = Read (\iota _T)$ and $Write (\iota ) = Write (\iota _T)$. Second, it restricts the target field holes (line 9) to use the source fields with the matching field write set, i.e., the hole for a target field $f_T$ uses the source field *f* when $Write (\iota _T, f_t) = Write (\iota , f)$. For example, given the sets from Fig. 5, the RW instruction holes for addi32 exclude sb but include lui, and the field holes for lui use only the $dst$ and $imm$ source fields. More generally, the RW sketch for addi32 consists of register-register instructions over $dst$ and $imm$, as intended. This sketch includes $2^{290}$ programs of length $k=5$ and depth $d\le 3$, resulting in a $2^{60}$ fold reduction in the size of the search space compared to the Naive sketch of the same length and depth.

### Generating Pre-load Sketches

**Fig. 6.**
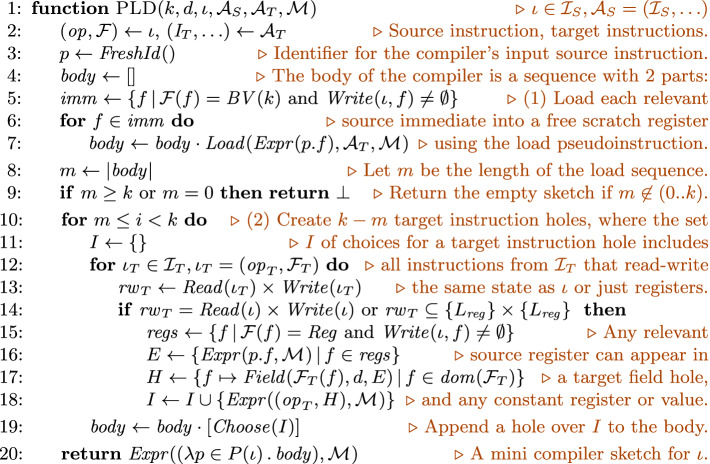
Pre-load sketch of length *k* and maximum depth *d* for $\iota$, $\mathcal {A}_S$, $\mathcal {A}_T$, and $\mathcal {M}_{}$. The $Load (E, \mathcal {A}_T, \mathcal {M}_{})$ function returns a sequence of target instructions that load the immediate value described by the expression *E* into an unused scratch register; see Fig. 4 for descriptions of other helper functions.

The pre-load sketch, $\textsc {PLD} \,(k, d, \iota , \mathcal {A}_S, \mathcal {A}_T, \mathcal {M}_{})$, is based on the observation that hand-written JITs use macros or subroutines to generate frequently used target instruction sequences. For example, compiling a source instruction with immediate fields often involves loading the immediates into scratch registers, and hand-written JITs include a subroutine that generates the target instructions for performing these loads. The pre-load sketch shown in Fig. 6 mimics this structure.

In particular, PLD generates a sequence of *m* concrete instructions that load the (used) immediate fields of $\iota$, followed by a sequence of $k-m$ instruction holes. The instruction holes can refer to both the source registers (if any) and the scratch registers (via the arbitrary bitvector constants included in the *Field* holes). The function $Load ( Expr (p.f), \mathcal {A}_T, \mathcal {M}_{})$ returns a sequence of target instructions that load the immediate *p*.*f* into an unused scratch register. This function itself is synthesized by JitSynth using a variant of the RW sketch.

As an example, the pre-load sketch for addi32 consists of two $Load$ instructions (lui and addiw in the generated C code) and $k-2$ instruction holes. The holes choose among register-register instructions in toy RISC-V, and they can refer to the $dst$ register of addi32, as well as any scratch register. The resulting sketch includes $2^{100}$ programs of length $k = 5$ and depth $d\le 3$, providing a $2^{190}$ fold reduction in the size of the search space compared to the RW sketch.

### Solving Compiler Metasketches

JitSynth solves the metasketch $\textsc {CMS} (\iota , \mathcal {A}_S, \mathcal {A}_T, \mathcal {M}_{})$ by applying the host synthesizer to each of the generated sketches in turn until a mini compiler is found. If no mini compiler exists in the search space, this synthesis process runs forever. To check if a sketch $\mathcal {S}$ contains a mini compiler, JitSynth would ideally ask the host synthesizer to solve the following query, derived from Definitions 4–6:
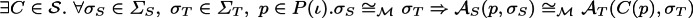


But recall that the state equivalence check $\cong _{\mathcal {M}_{}}$ involves universally quantified formulas over memory addresses and register names. In principle, these innermost quantifiers are not problematic because they range over finite domains (bitvectors) so the formula remains decidable. In practice, however, they lead to intractable SMT queries. We therefore solve a stronger soundness query (Definition 11) that pulls these quantifiers out to obtain the standard $\exists \forall$ formula with a quantifier-free body. The resulting formula can be solved with CEGIS [37], without requiring the underlying SMT solver to reason about quantifiers.

#### Definition 11 (Strongly Sound Mini Compiler)

Let $\mathcal {A}_S = (\mathcal {I}_S, \varSigma _S, \mathcal {T}_S, \varPhi _S)$ and $\mathcal {A}_T = (\mathcal {I}_T, \varSigma _T, \mathcal {T}_T, \varPhi _T)$ be two abstract register machines, $\cong _{\mathcal {M}_{}}$ an injective state equivalence relation on their states $\varSigma _S$ and $\varSigma _T$, and $C: P(\iota ) \rightarrow List (P(\mathcal {I}_T))$ a function for some $\iota \in \mathcal {I}_S$. We say that *C* is a strongly sound mini compiler for $\iota _{\mathcal {M}_{}}$ with respect to $\cong$ iff 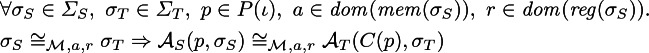 where $\cong _{\mathcal {M}_{},a,r}$ stands for the $\cong _{\mathcal {M}_{}}$ formula with *a* and *r* as free variables.

The JitSynth synthesis procedure is sound and complete with respect to this stronger query (Theorem 2). The proof follows from the soundness and completeness of the host synthesizer, and the construction of the compiler metasketch. We discharge this proof using Lean theorem prover [25].

#### Theorem 2

**(Strong soundness and completeness of** JitSynth**).** Let $\mathcal {C} = \textsc {CMS} (\iota , \mathcal {A}_S, \mathcal {A}_T, \mathcal {M}_{})$ be the compiler metasketch for the abstract instruction $\iota$, machines $\mathcal {A}_S$ and $\mathcal {A}_T$, and the state mapping $\mathcal {M}_{}$. If JitSynth terminates and returns a program *C* when applied to $\mathcal {C}$, then *C* is a strongly sound mini compiler for $\iota$ and $\mathcal {A}_T$ (soundness). If there is a strongly sound mini compiler in the most general search space $\{\textsc {Naive} (k, d, \iota , \mathcal {A}_S, \mathcal {A}_T, \mathcal {M}_{}) \,|\, k, d\in \mathbb {N}\}$, then JitSynth will terminate on $\mathcal {C}$ and produce a program (completeness).

## Implementation

We implemented JitSynth as described in Sect. 2 using Rosette [39] as our host language. Since the search spaces for different compiler lengths are disjoint, the JitSynth implementation searches these spaces in parallel [7]. We use $\varPhi (\mathbf {p}) = \texttt {length}(\mathbf {p})$ as the fuel function for all languages studied in this paper. This provides sufficient fuel for evaluating programs in these languages that are accepted by the OS kernel. For example, the Linux kernel requires eBPF programs to be loop-free, and it enforces this restriction with a conservative static check; programs that fail the check are not passed to the JIT [13].

## Evaluation

This section evaluates JitSynth by answering the following research questions:

- **RQ1**: Can JitSynth synthesize correct and performant compilers for real-world source and target languages?
- **RQ2**: How effective are the sketch optimizations described in Sect. 4?

**Fig. 7.**
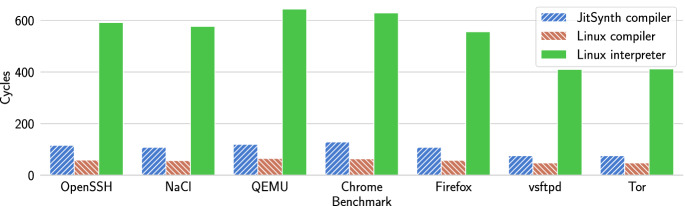
Execution time of eBPF benchmarks on the HiFive Unleashed RISC-V development board, using the existing Linux eBPF to RISC-V compiler, the JitSynth compiler, and the Linux eBPF interpreter. Measured in processor cycles.

### Synthesizing Compilers for Real-World Source-Target Pairs

To demonstrate the effectiveness of JitSynth, we applied JitSynth to synthesize compilers for three different source-target pairs: eBPF to 64-bit RISC-V, classic BPF to eBPF, and libseccomp to eBPF. This subsection describes our results for each of the synthesized compilers.

*eBPF to RISC-V.* As a case study, we applied JitSynth to synthesize a compiler from eBPF to 64-bit RISC-V. It supports 87 of the 102 eBPF instruction opcodes; unsupported eBPF instructions include function calls, endianness operations, and atomic instructions. To validate that the synthesized compiler is correct, we ran the existing eBPF test cases from the Linux kernel; our compiler passes all test cases it supports. In addition, our compiler avoids bugs previously found in the existing Linux eBPF-to-RISC-V compiler in Linux [27]. To evaluate performance, we compared against the existing Linux compiler. We used the same set of benchmarks used by Jitk [40], which includes system call filters from widely used applications. Because these benchmarks were originally for classic BPF, we first compile them to eBPF using the existing Linux classic-BPF-to-eBPF compiler as a preprocessing step. To run the benchmarks, we execute the generated code on the HiFive Unleashed RISC-V development board [35], measuring the number of cycles. As input to the filter, we use a system call number that is allowed by the filter to represent the common case execution.

Figure 7 shows the results of the performance evaluation. eBPF programs compiled by JitSynth JIT compilers show an average slowdown of $1.82\times$ compared to programs compiled by the existing Linux compiler. This overhead results from additional complexity in the compiled eBPF jump instructions. Linux compilers avoid this complexity by leveraging bounds on the size of eBPF jump offsets. JitSynth-compiled programs get an average speedup of $5.24\times$ compared to interpreting the eBPF programs. This evidence shows that JitSynth can synthesize a compiler that outperforms the current Linux eBPF interpreter, and nears the performance of the Linux compiler, while avoiding bugs.

**Fig. 8.**
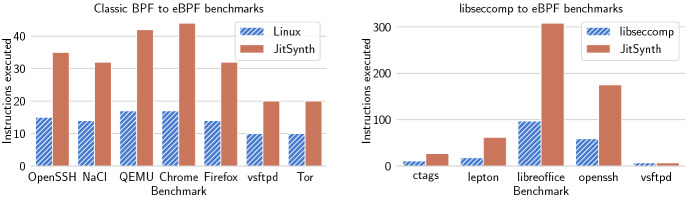
Performance of code generated by JitSynth compilers compared to existing compilers for the classic BPF to eBPF benchmarks (left) and the libseccomp to eBPF benchmarks (right). Measured in number of instructions executed.

*Classic BPF to eBPF.* Classic BPF is the original, simpler version of BPF used for packet filtering which was later extended to eBPF in Linux. Since many applications still use classic BPF, Linux must first compile classic BPF to eBPF as an intermediary step before compiling to machine instructions. As a second case study, we used JitSynth to synthesize a compiler from classic BPF to eBPF. Our synthesized compiler supports all classic BPF opcodes. To evaluate performance, we compare against the existing Linux classic-BPF-to-eBPF compiler. Similar to the RISC-V benchmarks, we run each eBPF program with input that is allowed by the filter. Because eBPF does not run directly on hardware, we measure the number of instructions executed instead of processor cycles.

Figure 8 shows the performance results. Classic BPF programs generated by JitSynth compilers execute an average of $2.28\times$ more instructions than those compiled by Linux.

*Libseccomp to eBPF.* libseccomp is a library used to simplify construction of BPF system call filters. The existing libseccomp implementation compiles to classic BPF; we instead choose to compile to eBPF because classic BPF has only two registers, which does not satisfy the assumptions of JitSynth. Since libseccomp is a library and does not have distinct instructions, libseccomp itself does not meet the definition of an abstract register machine; we instead introduce an intermediate libseccomp language which does satisfy this definition. Our full libseccomp to eBPF compiler is composed of both a trusted program to translate from libseccomp to our intermediate language and a synthesized compiler from our intermediate language to eBPF.

To evaluate performance, we select a set of benchmark filters from real-world applications that use libseccomp, and measure the number of eBPF instructions executed for an input the filter allows. Because no existing compiler exists from libseccomp to eBPF directly, we compare against the composition of the existing libseccomp-to-classic-BPF and classic-BPF-to-eBPF compilers.

Figure 8 shows the performance results. libseccomp programs generated by JitSynth execute $2.61\times$ more instructions on average compared to the existing libseccomp-to-eBPF compiler stack. However, the synthesized compiler avoids bugs previously found in the libseccomp-to-classic-BPF compiler [16].

**Fig. 9.**
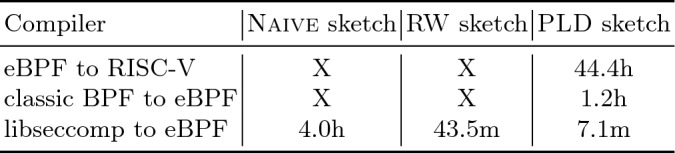
Synthesis time for each source-target pair, broken down by set of optimizations used in the sketch. An X indicates that synthesis either timed out or ran out of memory.

### Effectiveness of Sketch Optimizations

In order to evaluate the effectiveness of the search optimizations described in Sect. 4, we measured the time JitSynth takes to synthesize each of the three compilers with different optimizations enabled. Specifically, we run JitSynth in three different configurations: (1) using Naive sketches, (2) using RW sketches, and (3) using PLD sketches. For each configuration, we ran JitSynth with a timeout of 48 hours (or until out of memory). Figure 9 shows the time to synthesize each compiler under each configuration. Note that these figures do not include time spent computing read and write sets, which takes less than 11 min for all cases. Our results were collected using an 8-core AMD Ryzen 7 1700 CPU with 16 GB memory, running Racket v7.4 and the Boolector [29] solver v3.0.1-pre.

When synthesizing the eBPF-to-RISC-V compiler, JitSynth runs out of memory with Naive sketches, reaches the timeout with RW sketches, and completes synthesis with PLD sketches. For the classic-BPF-to-eBPF compiler, JitSynth times out with both Naive sketches and RW sketches. JitSynth only finishes synthesis with PLD sketches. For the libseccomp-to-eBPF compiler, all configurations finish, but JitSynth finishes synthesis about $34\times$ times faster with PLD sketches than with Naive sketches. These results demonstrate that the techniques JitSynth uses are essential to the scalability of JIT synthesis.

## Related Work

*JIT Compilers for In-kernel Languages.* JIT compilers have been widely used to improve the extensibility and performance of systems software, such as OS kernels [8, 11, 12, 26]. One notable system is Jitk [40]. It builds on the CompCert compiler [20] to compile classic BPF programs to machine instructions. Both Jitk and CompCert are formally verified for correctness using the Coq interactive theorem prover. Jitk is further extended to support eBPF [36]. Like Jitk, JitSynth provides formal correctness guarantees of JIT compilers. Unlike Jitk, JitSynth does not require developers to write either the implementation or proof of a JIT compiler. Instead, it takes as input interpreters of both source and target languages and state-mapping functions, using automated verification and synthesis to produce a JIT compiler.

An in-kernel extension system such as eBPF also contains a *verifier*, which checks for safety and termination of input programs [13, 40]. JitSynth assumes a well-formed input program that passes the verifier and focuses on the correctness of JIT compilation.

*Synthesis-Aided Compilers.* There is a rich literature that explores generating and synthesizing peephole optimizers and superoptimizers based on a given ISA or language specification [4, 9, 14, 17, 23, 33, 34]. Bansal and Aiken described a PowerPC-to-x86 binary translator using peephole superoptimization [5]. Chlorophyll [31] applied synthesis to a number of compilation tasks for the GreenArrays GA144 architecture, including code partitioning, layout, and generation. JitSynth bears the similarity of translation between a source-target pair of languages and shares the challenge of scaling up synthesis. Unlike existing work, JitSynth synthesizes a *compiler* written in a host language, and uses compiler metasketches for efficient synthesis.

*Compiler Testing.* Compilers are complex pieces of software and are known to be difficult to get right [22]. Recent advances in compiler testing, such as Csmith [41] and EMI [42], have found hundreds of bugs in GCC and LLVM compilers. Alive [19, 21] and Serval [28] use automated verification techniques to uncover bugs in the LLVM’s peephole optimizer and the Linux kernel’s eBPF JIT compilers, respectively. JitSynth complements these tools by providing a correctness-by-construction approach for writing JIT compilers.

## Conclusion

This paper presents a new technique for synthesizing JIT compilers for in-kernel DSLs. The technique creates per-instruction compilers, or compilers that independently translate single source instructions to sequences of target instructions. In order to synthesize each per-instruction compiler, we frame the problem as search using compiler metasketches, which are optimized using both read and write set information as well as pre-synthesized load operations. We implement these techniques in JitSynth and evaluate JitSynth over three source and target pairs from the Linux kernel. Our evaluation shows that (1) JitSynth can synthesize correct and performant compilers for real in-kernel languages, and (2) the optimizations discussed in this paper make the synthesis of these compilers tractable to JitSynth. As future in-kernel DSLs are created, JitSynth can reduce both the programming and proof burden on developers writing compilers for those DSLs. The JitSynth source code is publicly available at https://github.com/uw-unsat/jitsynth.
